# Green Method for the Preparation of Durable Superhydrophobic Antimicrobial Polyester Fabrics with Micro-Pleated Structures

**DOI:** 10.3390/molecules29061219

**Published:** 2024-03-08

**Authors:** Ying Zhao, Kaihong Chen, Jiehui Zhu, Huajie Chen, Yong Xia, Minglin Xu, Liyun Xu, Lirong Yao

**Affiliations:** 1 College of Textile and Clothing, Nantong University, Nantong 226019, China; zoey17715945532@163.com (Y.Z.);; 2 Langfang Feize Composites Technology Co., Ltd., Langfanng 065003, China; 3 National & Local Joint Engineering Research Center of Technical Fiber Composites for Safety and Protection, Nantong University, Nantong 226019, China

**Keywords:** plasma, micro-pleated structure, superhydrophobicity, antimicrobial property, water-soluble nanosilver

## Abstract

To produce functional protective textiles with minimal environmental footprints, we developed durable superhydrophobic antimicrobial textiles. These textiles are characterized by a micro-pleated structure on polyester fiber surfaces, achieved through a novel plasma impregnation crosslinking process. This process involved the use of water as the dispersion medium, water-soluble nanosilver monomers for antimicrobial efficacy, fluorine-free polydimethylsiloxane (PDMS) for hydrophobicity, and polyester (PET) fabric as the base material. The altered surface properties of these fabrics were extensively analyzed using scanning electron microscopy (SEM), Fourier transform infrared spectroscopy (FTIR), X-ray photoelectron spectrometry (XPS), thermogravimetric analysis (TGA), and water contact angle (WCA) measurements. The antimicrobial performance of the strains was evaluated using Gram-negative *Escherichia coli* and Gram-positive Staphylococcus aureus. After treatment, the fabrics exhibited enhanced hydrophobic and antimicrobial properties, which was attributed to the presence of a micro-pleated structure and nanosilver. The modified textiles demonstrated a static WCA of approximately 154° and an impressive 99.99% inhibition rate against both test microbes. Notably, the WCA remained above 140° even after 500 washing cycles or 3000 friction cycles.

## 1. Introduction

In contemporary complex living environments, there is an escalated emphasis on health, leading to a growing demand for hygiene and safety in textiles [1,2]. Porous textiles, which are susceptible to absorbing metabolic substances excreted by the human body, such as sweat and sebum, establish an environment conducive to the proliferation of bacteria and microorganisms. The presence of these microorganisms can result in issues such as clothing mold, disease transmission, and wound infections [3,4,5]. Notably, antimicrobial textiles have gained considerable attention in the context of human health protection and healthcare, due to their pivotal role in inhibiting the growth and reproduction of microorganisms, including bacteria and fungi [6,7,8]. Furthermore, they play an indispensable role in reducing the incidence of infectious diseases [9].

A biofilm is a community of microorganisms characterized by bacterial cells that are irreversibly attached to a substratum or interface, or to each other, embedded in a matrix of extracellular polymeric substances that they have produced [10,11]. Researchers have observed that biofilm formation follows a sequential colonization, from early colonizers such as streptococci, to bacterial proliferation, and to the final formation of biofilm [12], in which surface properties, such as morphology, chemistry, and surface energy, are the main factors affecting interfacial interactions [13,14]. According to the Cassie–Baxter wettability model, superhydrophobic surfaces (characterized by a water contact angle (WCA) of ≥ 150° and sliding angle SA of ≤ 10°) exhibit non-wetting, anti-fouling, and self-cleaning properties, resulting from the combination of low surface energy and surface roughness [15,16]. For many medical and industrial applications, superhydrophobic functional surfaces can effectively reduce the adhesion force between bacteria and material surface, hinder initial bacterial adherence, and inhabit biofilm formation, thus robustly resisting the spread and proliferation of bacteria [17,18,19]. Consequently, incorporating superhydrophobic surfaces with self-cleaning and anti-fouling functionalities has become a pivotal approach in the design of antimicrobial materials, with the aim of preventing bacterial growth [20,21].

Generally speaking, bacterial adherence can be explained by Derjaguin, Verwey, Landau, and Overbeek (DVLO) forces, which include electrostatic interaction, van der Waal forces, and steric interaction [22]. Researchers fabricate fabrics with dual-function hydrophobic and bactericidal coatings by thoroughly selecting highly active, oxidation-resistant, antimicrobial, and antiviral finishing agents. These agents are then integrated into superhydrophobic materials to reduce the viability and quantity of bacteria adhering to the surface [23]. If the bacteria come into contact with the surface or partly adhere to it, the bactericidal additives contained on the coatings will play an important role in killing the bacteria on contact and in adherence, by way of releasing additives and contact-killing; that is, showing bactericidal properties [19]. This approach not only impedes the initial adherence of bacteria but also actively eliminates bacteria and microorganisms that persistently adhere to the material surface. Ye et al. [24] combined fluorinated mesoporous silica nanoparticles (F-MSNs) and quaternary ammonium-functionalized silica nanoparticles (Q-MSNs) on various textile surfaces using a one-step dip-coating and spray-coating process, with polydimethylsiloxane (PDMS) as the binder. This property conferred superhydrophobicity and antimicrobial properties to the textile surfaces. The WCA was reduced from 152 ± 2° to 148 ± 2°, and the antimicrobial rate remained approximately 90% after 50 standard washing cycles. Additionally, after 600 sandpaper abrasion cycles, the contact angle reached approximately 143°, and the inhibition rates of *E. coli* and *S. aureus* were reduced to 82.9% and 81.3%, respectively. Pakdel et al. [25] developed cotton fabrics with superhydrophobic, antimicrobial, ultraviolet protection, and photothermal properties by dispersing PDMS and silver nanoparticles (Ag NPs) in an isopropanol solution. They then impregnated the fabrics and cured them at high temperature. The concentration of Ag NPs played a pivotal role in enhancing the antimicrobial activity of the fabrics, and the coating maintained a high contact angle of 168.52° after five accelerated washing cycles. Even after undergoing 1000 Martindale abrasion cycles, the WCA remained above 158°. However, under more rigorous abrasion conditions (0.9 kg load) or after 400 coarse wool fabric abrasion cycles and 15 sandpaper abrasion cycles, the superhydrophobic properties of the fabrics were significantly compromised.

Presently, the commonly employed methods for fabricating superhydrophobic surfaces include solution impregnation [26], layer-by-layer self-assembly [27], sol-gel [28], plasma [29,30], and hybrids of several approaches [31]. Recent research indicates that plasma treatment methods surpass conventional techniques in terms of efficiency. Specifically, the utilization of plasma discharge to facilitate surface etching, surface grafting, and surface crosslinking polymerization of materials, using energetic particles in a fully dry-state environment leads to the creation of low surface energy coatings and the formation of micro- and nano-roughness structures. This process culminates in the achievement of durable high-performance superhydrophobic surfaces [32,33].

Drawing inspiration from the periodic wrinkled skin structure of earthworms, Xu et al. [34] employed soft materials (i.e., PDMS) and induced a crosslinking gradient through Ar plasma treatment to generate uniform folds on fabric fibers. The pleated structure, coupled with the reversible adaptation property of PDMS, effectively handled the mechanical deformation of the superhydrophobic coating. The experimental results demonstrated that the superhydrophobic fabric retained its superhydrophobic properties, even after enduring 1000 friction cycles (44.8 kPa) and 800 standard washes. In contrast, traditional superhydrophobic surfaces encounter challenges related to complex production techniques and prolonged processing durations. Additionally, the synthesis or functionalization of nanoparticles relies on organic solvents such as tetrahydrofuran, methanol, and n-hexadecane, leading to increased production costs and significant environmental pollution.

To address the environmental concerns associated with the superhydrophobic preparation process, Zhang et al. [35] devised an eco-friendly approach based on a water-based solution. This method involved the utilization of alumina particles to construct rough microstructures, along with fluorosurfactants and fluorinated alkyl silanes as low surface energy materials. The resulting coatings exhibited/water repellency on diverse substrates, including glass, wood, fabrics, and metals, with WCAs exceeding 150°. Ge et al. [36] innovatively treated PDMS with oxygen plasma and subsequently dispersed it uniformly in water via ultrasonication to create an oil-in-water emulsion. This emulsion was employed to form an intelligent self-healing and robust superhydrophobic coating on cotton fibers, which was achieved without the use of organic solvents or fluorine-based components. After 25 washing cycles, the WCA exhibited a minimal decrease, remaining above 150°. However, following 10 abrasion cycles, the fabric lost its superhydrophobic properties.

**Scheme 1:**
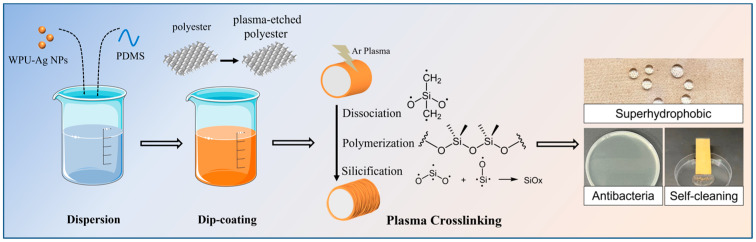
Schematic diagram of the process of preparing robust and durable superhydrophobic antimicrobial fabrics with micro-pleated structures using green methods.

In this study, we developed superhydrophobic antimicrobial polyester fabrics through a combination of environmentally friendly low-temperature plasma technology and a water-based treatment approach. Our approach involved the use of chemically reduced water-soluble nanosilver in situ as an antimicrobial agent and fluorine-free polydimethylsiloxane (PDMS) as a hydrophobic agent. This strategic amalgamation not only conferred enduring superhydrophobic and antimicrobial characteristics to the fabrics but also prioritized comfortable wearability. As illustrated in Scheme 1, the PDMS/Ag–water emulsion was firstly configured, the polyester fiber surface underwent plasma surface etching to increase the roughness prior to impregnation, followed by the low temperature plasma-induced crosslinking to create micro-pleated structures on the polyester fabric surface. These structures endow the fabrics with desirable superhydrophobic antimicrobial properties. The primary objective of this study was to develop a straightforward, efficient, and environmentally friendly method for producing robust superhydrophobic antimicrobial polyester fabrics. The results unequivocally demonstrated that the prepared superhydrophobic antimicrobial functional fabrics exhibited exceptional superhydrophobicity and antimicrobial properties. When the WCA reached approximately 154° and the nanosilver loading was greater than or equal to 150 mg/kg, the fabric maintained a remarkable antibacterial rate of 99.99%, even after 100 washing cycles.

## 2. Results and Discussion

### 2.1. Preparation and Characterization of Polydimethylsiloxane (PDMS) Composite Emulsion

PDMS is a water-insoluble polymer. In this process, we selected low molecular-weight PDMS and blended it with water using a high-speed shear emulsifying machine. The PDMS molecules underwent a series of intense mechanical and hydraulic shearing actions within the precision gap of the stator rotor. This process resulted in uniform and fine emulsification, fragmentation, and instantaneous dissolution of the incompatible substances, forming an oil-in-water homogeneous emulsion. The resulting finishing solution exhibited translucency, and upon microscopic examination (Figure S1a), the oil droplets were granularly dispersed in the water. The zeta potential at the end of the dispersion process was measured at −33.6 mV (Figure S1b). The average particle size of the dispersed PDMS emulsion was approximately 835.31 nm, and even after 30 min of standing, it remained at approximately 954.11 nm (Figure S1c,d). These tests of potential and particle size confirmed that the emulsion maintained a certain degree of stability over a specified period, making it suitable for use in the post-impregnation treatment of fabrics.

**Figure 1:**
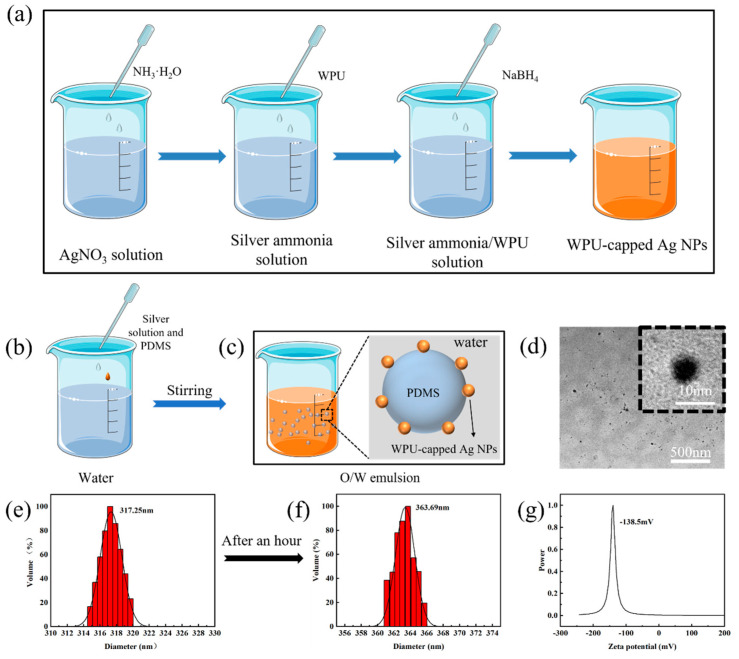
(**a**) Schematic diagram of the preparation process of the nanosilver solution. (**b**) Schematic diagram of the preparation of the PDMS/Ag-water emulsion. (**c**) Dispersion state of PDMS and WPU-capped Ag in water. (**d**) TEM image of the PDMS/Ag-water emulsion. (**e**,**f**) Particle size distribution of the PDMS/Ag-water emulsion before and after one hour of resting at room temperature. (**g**) Zeta potential of the PDMS/Ag-water emulsion.

Due to its low adhesion and poor dispersion, nanosilver necessitates encapsulation within a polymer carrier material to maintain its antibacterial efficacy [37]. In this study, we prepared aqueous nanosilver adhesive solutions with high dispersion, stability, and adhesion properties using waterborne polyurethane (WPU) as a protective agent. The process for preparing the nanosilver aqueous solution is depicted in Figure 1a. Subsequently, the nanosilver solution was diluted in deionized water according to the desired nanosilver loading, and the PDMS polymer was added and dispersed through high-speed homogeneous shear dispersion (Figure 1b,c). The TEM results in Figure 1d demonstrated the uniform dispersion of the PDMS polymer and generated Ag nanomonomers in water. The inset at the top right shows Ag monomers with a particle size of approximately 9.7 nm, consistent with the findings in the literature [38], confirming the successful preparation of silver nanoparticles. The particle size of the PDMS/Ag–water emulsion was approximately 317.25 nm, and after approximately one hour of standing, it slightly increased to 363.69 nm due to the movement of oil droplet molecules (Figure 1e,f).

Colloidal solution stability can be assessed through the absolute value of the zeta potential, and it is established that when the absolute value of the zeta potential exceeds 40 mV, the emulsion is considered to possess good stability. As depicted in Figure 1g, the measured zeta potential of the emulsion was approximately −132 mV. Compared to those of the PDMS–water emulsion, the PDMS/Ag–water emulsion exhibited a smaller particle size and greater potential. This difference can be attributed to the fact that aqueous polyurethanes containing amide groups tightly adsorb on the surface of the Ag nanoparticles during preparation, forming polyurethane-modified Ag colloidal particles. These particles are shared at the interface of two phases (aqueous oil) and bridge them. In some instances, colloidal particles can act as surfactants in dispersed emulsions, contributing to the excellent stability of PDMS/Ag oil-in-water emulsions, similar to Pickering emulsions, in which ultrafine solid particles serve as emulsifiers [39,40].

### 2.2. Preparation and Characterization of Superhydrophobic Antimicrobial Fabrics

**Figure 2:**
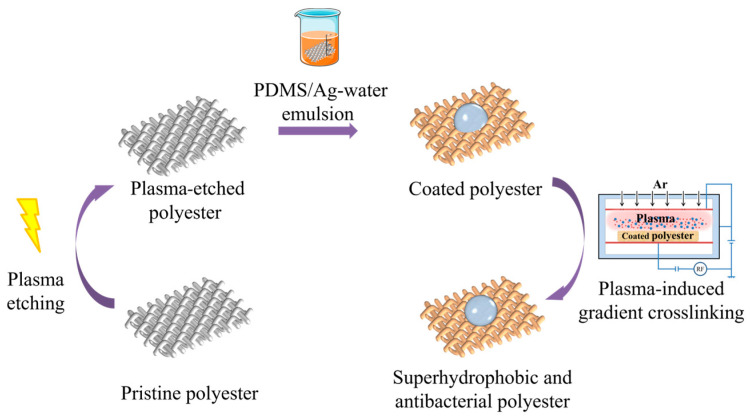
Schematic diagram of the preparation of the PDMS/Ag@PET superhydrophobic antimicrobial polyester fabric.

The process for the further preparation of superhydrophobic antimicrobial polyester fabrics using the PDMS/Ag-water emulsion is detailed in Figure 2. Initially, the polyester fabrics underwent plasma etching. Subsequently, the fabrics were immersed in the finishing solution for 30 min. The polyester fabrics with various nanosilver loadings were prepared by controlling the roll-over rate to 100% and then drying at 60 °C. Finally, low-pressure plasma crosslinking was employed to create superhydrophobic antimicrobial polyester fabrics with nanosilver encapsulated in PDMS. Additionally, PDMS@PET fabrics were prepared without the addition of the nanosilver solution solely by impregnating the fabrics with a PDMS-water emulsion following the same preparation process as described above.

**Figure 3:**
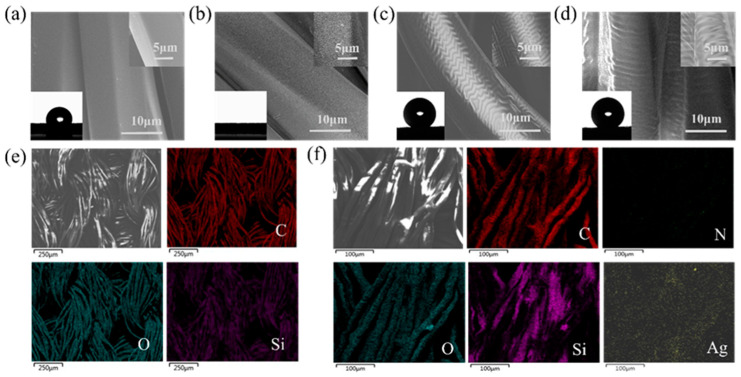
SEM images of (**a**) pristine polyester fabric, (**b**) plasma-etched polyester fabric, (**c**) PDMS@PET fabric, and (**d**) PDMS/Ag@PET fabric, with magnified views of the fiber surface in the upper right corner and photographs of the corresponding WCA in the lower left corner. Elemental mapping of (**e**) PDMS@PET fabric and (**f**) PDMS/Ag@PET fabric images.

Polyester fibers, like most synthetic fibers, possess inherent hydrophobicity. Therefore, enhancing the wetting properties of these materials before impregnation is essential. Air plasma etching introduces a multitude of oxygen-containing or other polar groups on the polymer surface, serving as binding sites to enhance the cohesion between nanoparticles, fabric, and other substances. This process improved the strength between the substrate and nanoparticles, PDMS, and other materials. Furthermore, it significantly enhances the surface roughness and specific surface area of the fabric [41,42]. As depicted in the SEM images in Figure 3a,b, compared with the original polyester fabric, the etched fabric exhibited dense and uniform grooves on the surface. Plasma energy bombardment induces chemical bond changes in polyester fiber molecules, including the breaking, polymerization, and reorganization of bonds such as C-C, C-O, and C=O. These changes alter the surface morphology. The inset in the bottom left of the figure reveals that the contact angle of the original polyester fabric was approximately 70°, whereas the contact angle of the etched polyester fabric approached 0°, indicating significantly improved hydrophilicity. When both the untreated and etched fabrics were placed in water simultaneously, the untreated fabric remained afloat, while the etched fabric began to sink within approximately 1 s. Within approximately 9 s, the untreated fabric slowly wetted the water surface, whereas the etched fabric had already sunk to the bottom of the reagent bottle (Figure S2a). An XPS analysis of both the untreated and plasma-etched fabrics revealed a substantial increase in the O1s peak after plasma etching, with the atomic content increasing from 29.14% to 39.24%. Conversely, the C1s peak intensity significantly decreases as the atomic content decreases from 69.77% to 59.14%. Split-peak fitting of C demonstrated a considerable decrease in the C-C group content, while the C-O group content remained relatively stable, and the C=O element content increased (Figure S2b–d and Table S1). These findings further confirm the enhanced wettability of the etched polyester fabrics.

Furthermore, SEM images (Figure 3c,d) reveal that plasma impregnation crosslinking results in a distinct folded structure on the surface of the fabric, accompanied by a significant improvement in surface roughness. This microfold structure plays a pivotal role in the development of superhydrophobic properties in fabrics. Static WCA images of the PDMS@PET and PDMS/Ag@PET fabrics demonstrated contact angles of approximately 154°, indicating that the addition of nanosilver did not hinder graft polymerization of the PDMS film on the surface of the fabric. Additionally, the EDS elemental mapping results of the PDMS-coated crosslinked fabrics confirmed the presence of three elements, namely C, O, and Si, with Si originating exclusively from PDMS. The PDMS/Ag@PET fabric additionally contained N and Ag, which are attributed to the WPU shells and silver monomers that result from the chemical reduction of AgNO_3_. These results provide evidence of successful loading of PDMS and Ag on the polyester fabric surface (Figure 3e,f).

**Figure 4:**
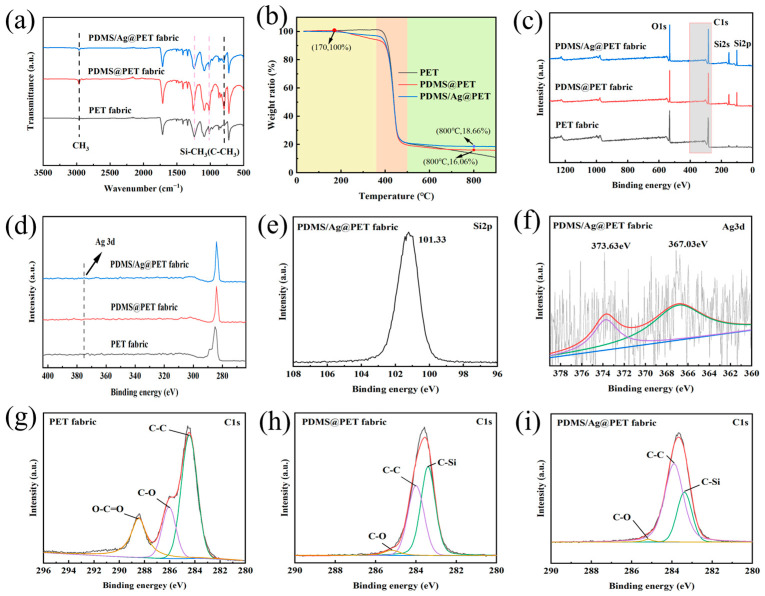
(**a**) FTIR, (**b**) TGA, and (**c**) XPS data of pristine polyester, PDMS@PET, and PDMS/Ag@PET fabrics. (**d**) Magnification of the area framed in (**c**). High-resolution (**e**) Si 2p and (**f**) Ag 3d spectra of the PDMS/Ag@PET fabrics. High-resolution C1s spectra of the pristine polyester fabric (**g**), PDMS@PET fabric (**h**), and PDMS/Ag@PET fabric (**i**).

The chemical structure of the fabrics was analyzed based on the FTIR spectra presented in Figure 4a. Characteristic peaks corresponding to C-O and C-C stretching vibrations at 1240 cm^−1^ and 1020 cm^−1^, respectively, were observed, which are characteristic of pristine PET fabrics. However, in the PDMS@PET and PDMS/Ag@PET fabrics, new characteristic peaks emerged. Notably, a distinctive Si-CH_3_/(C-CH_3_) peak appeared at 798 cm^−1^, along with an enhanced methyl peak at 2970 cm^−1^ (Figure 4a) [43,44,45]. These observations suggest that PDMS was successfully grafted onto the surface of the PET fibers.

The thermal stability of PDMS and nanosilver surpasses that of polyethylene terephthalate under oxygen-free conditions, making thermogravimetric analysis a valuable tool for characterizing fabric heat resistance. In Figure 4b, the mass loss pattern of the fabrics with increasing temperature is illustrated, revealing three distinct weight loss processes under a nitrogen atmosphere. The first stage involves the initial decomposition of the original polyester and surface crosslinked polyester fabric. Raw polyester initially decomposes at temperatures ranging from 30 °C to 356 °C, with minimal mass loss. Since the initial decomposition temperature of the PDMS film is 170 °C, both the PDMS@PET and PDMS/Ag@PET fabrics begin to decompose at this point, with a gradual loss of mass. The difference in fabric mass loss between PDMS@PET and PDMS/Ag@PET at this stage arises from the superior thermal stability of the silver nanoparticles, which results in relatively slower degradation of the PDMS/Ag@PET fabric. In the second stage, occurring between 356 °C and 501 °C, poly(ethylene terephthalate) begins to decompose rapidly, releasing a significant volume of gas and resulting in rapid and substantial mass loss. In contrast, the PDMS@PET and PDMS/Ag@PET fabrics exhibited relatively high thermal stabilities during this stage. The third stage primarily represents the residual decomposition phase. At temperatures exceeding 501 °C, PET continues to degrade, gradually producing solid carbon compounds. The residues of the PDMS@PET and PDMS/Ag@PET fabrics also continued to degrade, albeit with a relatively high remaining content. This is because the PDMS monomer completely breaks down at 700 °C, and the final decomposition of residues is attributed to the SiOx material formed when silicon on the PDMS surface undergoes oxidation due to plasma action [45]. At this stage, the slight difference in residue content between the PDMS@PET and PDMS/Ag@PET fabrics (2.6%) can be attributed to the addition of inorganic nanosilver, which leads to an increase in residual content.

To further investigate the changes in the surface chemical composition of the PET fabrics before and after treatment, XPS analysis was performed. In addition to the C (284.08 eV) and O (531.08 eV) peaks present on pristine PET, the Si peak appeared on PDMS@PET, and both Ag and Si peaks were visible on PDMS/Ag@PET (Figure 4c). The characteristic peaks at 101.08 eV and 152.08 eV correspond to Si 2p and Si 2 s, respectively. Since the tested sample had a nanosilver loading of only 200 mg/kg, XPS detected a minute amount of Ag. Ag 3d exhibited a distinct peak at 375.08 eV, in accordance with the EDS results (Figure 4d). As shown in Figure 4f, the Ag 3d characteristic peak can further split into peaks at 373.63 eV and 367.03 eV [46].

Subsequently, changes in the Si content after plasma crosslinking treatment were investigated by splitting and fitting the C 1 s peaks. As depicted in Figure 4g–i, characteristic peaks of O-C=O, C-O, and C-C appeared at 288.48 eV, 286.08 eV, and 284.48 eV, respectively [47,48]. Crosslinking treatment led to a decrease in the relative contents of these three peaks, accompanied by the appearance of new characteristic peaks associated with C-Si at 283.40 eV in the spectrum of the treated fabric. This change can be attributed to the fact that plasma treatment opens more C-Si and Si-O bonds on the main and side chains of PDMS. High-energy particles generated by plasma bombardment also break down more C-C bonds and O-C=O bonds with lower bond energies on the main chain of PET, leading to the formation of C and O active sites. These sites subsequently undergo recombination and self-polymerization, resulting in stable grafting of the PDMS film onto the surface of the PET fibers. Consequently, the content of C-Si bonds increases. These results collectively indicate the successful adhesion of the PDMS-Ag nanocomposite coating to the fabric and the effectiveness of plasma in enhancing PDMS graft polymerization on the PET surface [49].

### 2.3. Hydrophobicity and Wettability Resistance of Superhydrophobic Antimicrobial Fabric

**Figure 5:**
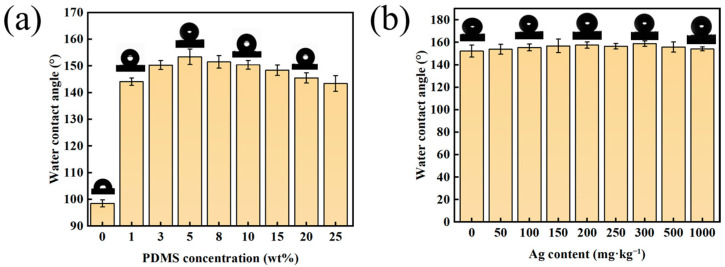
Variation in WCA with respect to the (**a**) PDMS concentration for the PDMS@PET fabrics and (**b**) nanosilver content for the PDMS/Ag@PET fabrics at a PDMS concentration of 5 wt.%.

The hydrophobic properties of the treated polyester fabrics were thoroughly investigated. In Figure 5a, it is evident that the static WCA of the fabric exhibited a pattern of initial increase and subsequent decrease with increasing PDMS concentration. When the PDMS concentration reached approximately 5 wt.%, the contact angle of the PDMS@PET fabrics reached 153.6°. Notably, for the PDMS/Ag@PET fabrics, when the PDMS concentration was maintained at this specific level (5 wt.%), changes in the contact angle tended to stabilize as the nanosilver content increased. This observation, when considered alongside Figure S3, reveals that in the absence of PDMS, fabrics with only the addition of nanosilver (Ag@PET) maintained hydrophobicity with increasing silver content. This was attributed to the presence of a small quantity of water-soluble polyurethane-modified nanosilver particles on the fiber surface. Notably, PDMS played a pivotal role in conferring superhydrophobicity to the fabric. In other words, the addition of water-soluble nanosilver had no adverse effect on the superhydrophobicity of the PDMS@PET fabrics.

Further investigations focused on how the PDMS concentration influenced hydrophobicity, and scanning electron microscopy (SEM) and atomic force microscope (AFM) were also conducted. The results indicate that, at a PDMS impregnation concentration of 1 wt.%, the hydrophobicity of the fiber surface improved due to the presence of a certain amount of surface energy, resulting in a contact angle of approximately 144°. However, superhydrophobicity was not achieved at this concentration, and it was challenging for PDMS to form a complete pleated structure on the surface of PET fibers. The measured Ra value was 9.11 nm. (Figure S4a,d).

As the PDMS concentration increased to 5 wt.%, the contact angle reached a peak of approximately 153°, and the SEM images revealed a distinct, wrinkled, thin film structure on the fiber surface. This corresponded to an improvement in surface roughness, with R_a_ value of 20.3 nm (Figure S4b,e). However, when the PDMS concentration exceeded a certain threshold (15 wt.%), the increased thickness of the PDMS on the fabric surface led to pronounced adhesion between the fibers. Consequently, it became difficult to create wrinkles on the fiber surface due to the limited depth of plasma penetration and the weakened crosslinking effect on PDMS. This resulted in a R_a_ value of 4.53 nm (Figure S4c,f). Despite the low surface energy of the fabric, the insufficient roughness led to a reduction in the hydrophobicity of the PET fabrics, which was consistent with the contact angle data. Therefore, the optimal superhydrophobicity of the prepared fabrics was achieved only at a suitable PDMS concentration. For comparison, PDMS was also dissolved in an ethanol solution. At the same PDMS concentration, the WCA of the modified polyester fabrics was 152.3°. With an increase in the PDMS concentration in the organic solvent solution, the WCA exhibited a similar pattern of initial increase and subsequent decrease, reaching a maximum of 157.4° when the concentration reached 20 wt.% (Figure S5). This suggests that the dispersion of PDMS in both water-based and organic solvent systems provides comparable superhydrophobicity to that of modified polyester fabrics.

**Figure 6:**
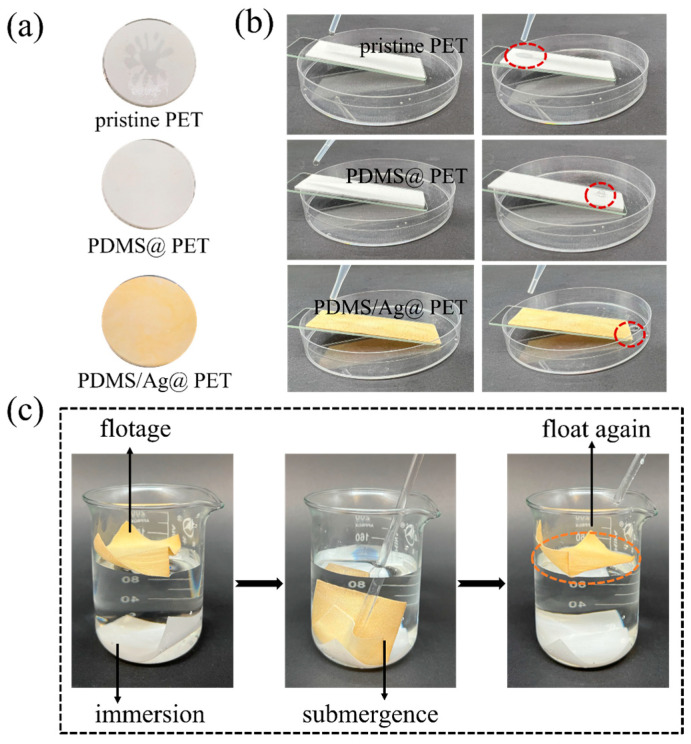
(**a**) Spray wettability, (**b**) slip of water droplets on different fabric slopes, and (**c**) wetting of virgin polyester fabric (white) and PDMS/Ag@PET fabric (yellow) in water (The red circles represent the state of the water droplet at the same moment, and the orange circle is used to highlight the final state of the fabric).

To assess the wetting resistance of the crosslinked fabrics, a series of experiments were conducted. As shown in Video S1 and Figure 6a, 250 mL of deionized water was sprayed onto the fabrics. It is evident that the polyester fabric was extensively wetted with a spray rating of only two. In contrast, when water droplets were applied to the superhydrophobic surfaces, the droplets quickly rolled off, reaching a spray rating of four–five. Further confirmation of these findings was obtained when different fabrics were placed at an angle in a Petri dish and deionized water was dripped from above (Figure 6b). The original polyester samples were swiftly wetted, while the droplets on the PDMS@PET and PDMS/Ag@PET fabrics rapidly descended to the bottom of the slide. These results were consistent with the spraying experiment, confirming the excellent anti-wetting performance of the crosslinked treated fabrics.

Additionally, untreated PET and PDMS/Ag@PET fabrics were simultaneously submerged in a beaker filled with water. Over time, the untreated fabric sank to the bottom due to prolonged soaking, while the PDMS/Ag@PET fabric remained afloat. Notably, when the PDMS/Ag@PET fabric was submerged in an external force, it exhibited a remarkable “silver mirror” phenomenon. Upon removal of the external force, the fabric was immediately reset, after which it was allowed to dry (Figure 6c). These observations can be attributed to the presence of a micro-pleated structure on the surface of the PDMS/Ag@PET fabrics, which facilitates the formation of an air layer between the liquid and the substrate. Consequently, the fabrics are less prone to infiltration, leading to these remarkable phenomena.

### 2.4. Antimicrobial Properties

**Figure 7:**
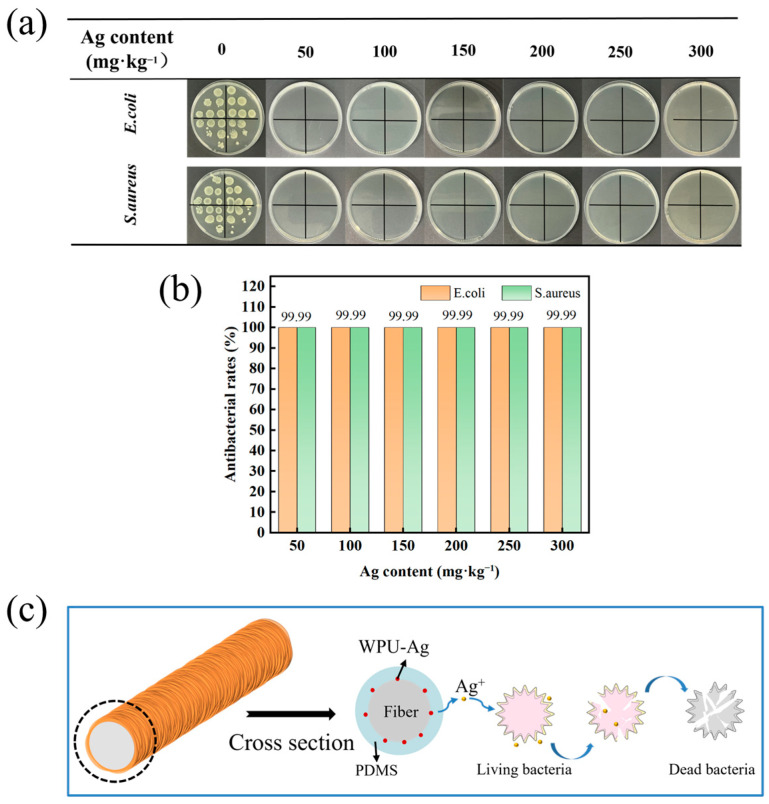
Antimicrobial properties of the PDMS/Ag@PET fabrics. (**a**) Photographs of *E. coli* and *S. aureus* colonies in nutrient agar Petri dishes. (**b**) Inhibition rate. (**c**) Schematic diagram of the nanosilver-releasing bactericidal process.

In the field of textiles, nanosilver has significant utility for imparting antibacterial and antiviral properties to fabrics. The colony distribution graph presented in Figure 7a illustrates that the original polyester fabric exhibited no inhibitory effect on *E. coli* or *S. aureus*. Furthermore, in the absence of added nanosilver particles, the PDMS-coated polyester fabric produced fewer bacterial colonies on the nutrient agar culture dish than the original polyester fabric did. The superhydrophobic surface’s antibacterial adhesion properties resulted in antibacterial rates of approximately 86.44% against *E. coli* and 88.93% against *S. aureus* (Figure S6). Remarkably, the prepared PDMS/Ag@PET fabric significantly inhibited both *E. coli* and *S. aureus*, with no bacterial colonies observed on nutrient agar Petri dishes. Silver-loaded polyester fabrics with silver loadings ranging from 50 to 300 mg/kg exhibited an impressive inhibition rate of 99.99% against both bacterial strains (Figure 7b). These results underscore the antimicrobial efficacy of the PDMS/Ag@PET fabrics.

The antimicrobial mechanism of the PDMS/Ag@PET fabrics is rooted in the encapsulation of silver nanoparticles within the PDMS coating. When in contact with bacteria, silver ions are released from the coating and gradually enter the external environment. In this environment, silver ions interact with bacterial cells, leading to the disruption of essential components within the bacteria and eventual bacterial death (Figure 7c). The antimicrobial action of silver ions includes four primary steps [19,50,51]. The first is interference with cell wall synthesis, as follows: the cell walls of both Gram-positive and Gram-negative bacteria contain peptidoglycan and lipopolysaccharide, which play pivotal roles in bacterial adhesion. Silver ion antimicrobial agents inhibit the crosslinking of polysaccharide chains with tetrapeptides, compromising the integrity of the cell wall and ultimately causing bacterial death. The second is cell membrane damage, as follows: upon contact with bacteria, silver ions are released and bind to proteins on the bacterial cell membrane. This interaction disrupts the structure of the bacterial cell membrane, leading to bacterial death. The third is inhibition of protein synthesis, as follows: silver ions can alter and halt the process of protein synthesis within bacterial cells. The fourth is interference with nucleic acid synthesis, as follows: silver nanoparticles, due to their small size and high specific surface area, can penetrate the interior of bacterial cells and impede the replication of genetic information, including the synthesis of DNA and RNA, as well as the transcription of mRNA from DNA templates.

### 2.5. Mechanical Robustness and Resistance to Acids and Alkalis

**Figure 8:**
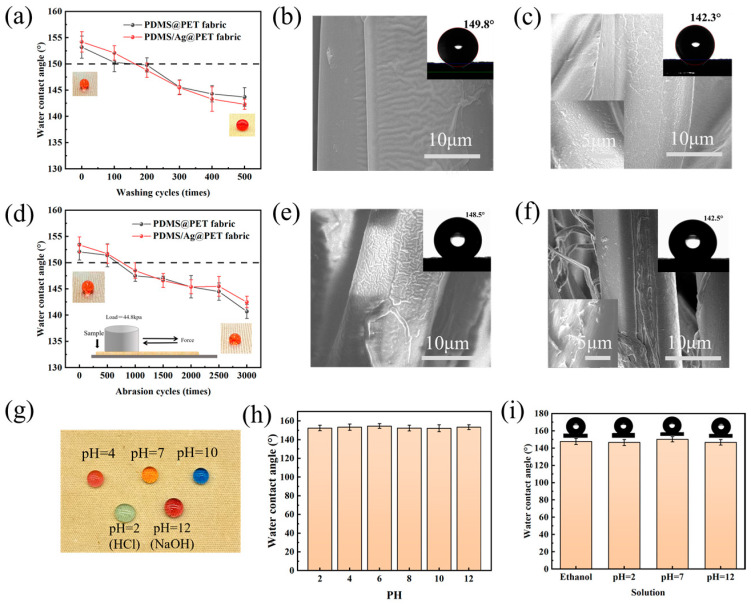
(**a**) Effect of the number of washing cycles on the WCA. SEM images and WCA photographs of PDMS/Ag@PET fabric washed (**b**) 200 times and (**c**) 500 times. (**d**) Effect of the number of abrasion cycles on the WCA. SEM images and CA photographs of PDMS/Ag@PET fabric abraded (**e**) 1000 times and (**f**) 3000 times. (**g**) Photographs of droplets on the surface of PDMS/Ag@PET fabrics at different pH values. (**h**) WCA of water droplet solutions (10 μL) of different pH values on PDMS/Ag@PET fabrics. (**i**) CA of PDMS/Ag@PET fabrics after 12 h of immersion in different solutions.

The PDMS@PET- and PDMS/Ag@PET-coated fabrics were subjected to a rigorous testing regime involving up to 500 wash cycles, and both exhibited remarkable resistance to washing. As depicted in Figure 8a, the static WCA gradually decreased with the number of wash cycles but consistently remained above 140° throughout the testing period. The decrease in the WCA was attributed to the gradual damage to the surface fold structure of the fabric caused by washing. After 200 washes, the WCA reached 149.8°, and some of the pleated membrane structures on the surface of the fabric showed signs of damage (Figure 8b), resulting in the loss of superhydrophobicity. After 500 washes, the contact angle decreased to 142.3°, and the surface of the PDMS membrane fold structure was nearly absent. However, residual membranes still covered the fiber surface (Figure 8c), enabling the PDMS/Ag@PET fabrics to maintain considerable hydrophobicity and ensure the durability of their hydrophobic properties.

The durability of the PDMS@PET and PDMS/Ag@PET fabrics under mechanical abrasion was also examined as a crucial criterion for practical applications. At a pressure load of 44.8 kPa, both types of fabrics displayed a decrease in WCA with an increasing number of abrasion cycles (Figure 8d). After 3000 wear cycles, the WCA remained above 140°, indicating good hydrophobicity. SEM analysis revealed that, after 1000 abrasion cycles, the microfold structure on the fabric surface underwent minimal damage, and a significant amount of the PDMS film remained, resulting in a high static WCA of 148.1° (Figure 8e). However, after 3000 abrasion cycles, the fold structure of the PDMS membrane almost disappeared, and the fiber surface experienced severe damage (Figure 8f), leading to a reduction in hydrophobicity. Photographs of water droplets on the fabric surface before and after 3000 abrasion cycles clearly demonstrate the change in droplet behavior (Figure 8g).

Furthermore, the superhydrophobic antimicrobial fabrics were tested in acidic and alkaline environments. Solutions of NaOH and HCl, as well as acidic and alkaline solutions with varying pH values adjusted using these reagents, were employed as test droplets. Figure 8g illustrates that the droplets maintained a spherical shape on the fabric surface and did not wet the fabric. Subsequently, droplets with pH values ranging from 2 to 12 were dropped onto the fabric samples at a controlled flow rate. The average WCA was determined after testing five different points on each sample, and the results consistently yielded CAs of approximately 150°, indicative of excellent liquid repellency (Figure 8h). The fabrics were further immersed in ethanol and solutions with pH values of 2, 7, and 12 for 12 h. The subsequent contact angles measured were 147.6°, 147.5°, 150.1°, and 146.6°, respectively (Figure 8i). Notably, the most significant decrease in contact angle under alkaline conditions may be attributed to the fact that PDMS contains silane blocks, akin to inorganic silica, which exhibit weak acidity and can react with strong alkalis to form corresponding silicates or other compounds, rendering the treated fabrics susceptible to alkaline environments.

### 2.6. Antimicrobial Durability

**Figure 9:**
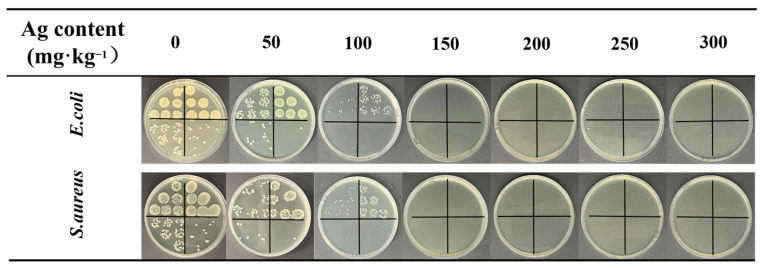
Photographs of *E. coli* and *S. aureus* colonies in nutrient agar Petri dishes after 100 standard washes of PDMS/Ag@PET fabrics.

To assess the durability of the nanosilver coating, the antimicrobial performance of polyester fabrics with silver contents ranging from 50 to 300 mg/kg was tested after subjecting them to 100 soaping cycles. The results, as illustrated in Figure 9 and summarized in Table 1, provide valuable insights into the long-term effectiveness of the nanosilver coating. After 100 washes of the fabric with a silver content of 50 mg/kg, the antibacterial capability against *E. coli* and *S. aureus* was 90.99% and 91.99%, respectively. The silver-loaded fabrics at 100 mg/kg maintained a high level of antibacterial activity, with inhibition rates of 98.89% against *E. coli* and 96.97% against *S. aureus* after 100 wash cycles.

When the silver content reached 150 mg/kg or higher, the antimicrobial activity remained at 99.99% after 100 washes. Notably, plasma etching of the polyester fabric surface resulted in enhanced adhesion of polyurethane-capped nanosilver monomers within the surface grooves, improving the overall adhesion of the polyester fabric. Additionally, the presence of the PDMS film protected the nanosilver during the washing process, leading to improved encapsulation and, consequently, better bacteriostatic washing resistance in the PDMS and silver-loaded polyester fabrics.

### 2.7. Self-Cleaning Properties

**Figure 10:**
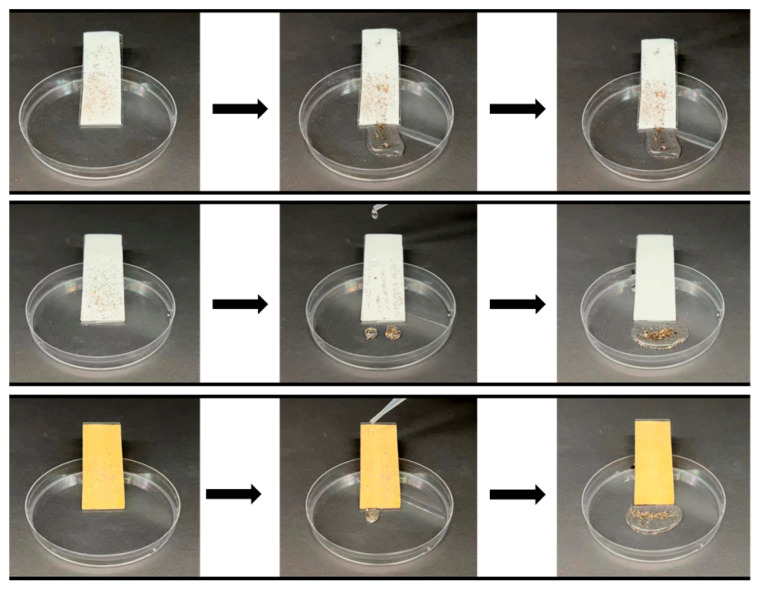
Self-cleaning process of virgin polyester fabrics, PDMS @PET fabrics, and PDMS/Ag@PET fabrics.

Self-cleaning performance is a pivotal characteristic of superhydrophobic surfaces. To evaluate the self-cleaning effect, dust and fine sand, common pollutants in everyday life, were used. These solid contaminants were randomly dispersed on the sample surfaces, which were securely positioned on slides. Deionized water (20 μL) was then allowed to drip onto the samples from above, simulating the action of a droplet. The results, depicted in Figure 10 and Video S2, provide clear evidence of the self-cleaning capabilities of the various fabric surfaces. On the original PET fabric surface, dust and fine sand swiftly adhered to the fabric along with the liquid droplets, leaving conspicuous traces of contamination. This demonstrated the absence of self-cleaning functionality on the original polyester fabric. Conversely, on the surfaces of the PDMS@PET and PDMS/Ag@PET fabrics, contaminants were easily swept away by the water droplets, restoring a clean surface with a self-cleaning effect. This observation underscores the higher water surface tension and lower surface energy of the PDMS-coated crosslinked fabrics. Importantly, the inclusion of nanosilver did not compromise the cleaning efficacy of the PDMS coating, which plays a pivotal role in preventing dust and pollution on the fabric.

### 2.8. Wearability of Superhydrophobic Antimicrobial Fabrics

To assess the effect of the plasma cross-linking treatment on fabric performance, an array of tests was conducted, including tests of tensile properties, air permeability, moisture permeability, and water penetration resistance, on both untreated virgin polyester fabrics and superhydrophobic antimicrobial fabrics produced through the plasma impregnation cross-linking method.

**Figure 11:**
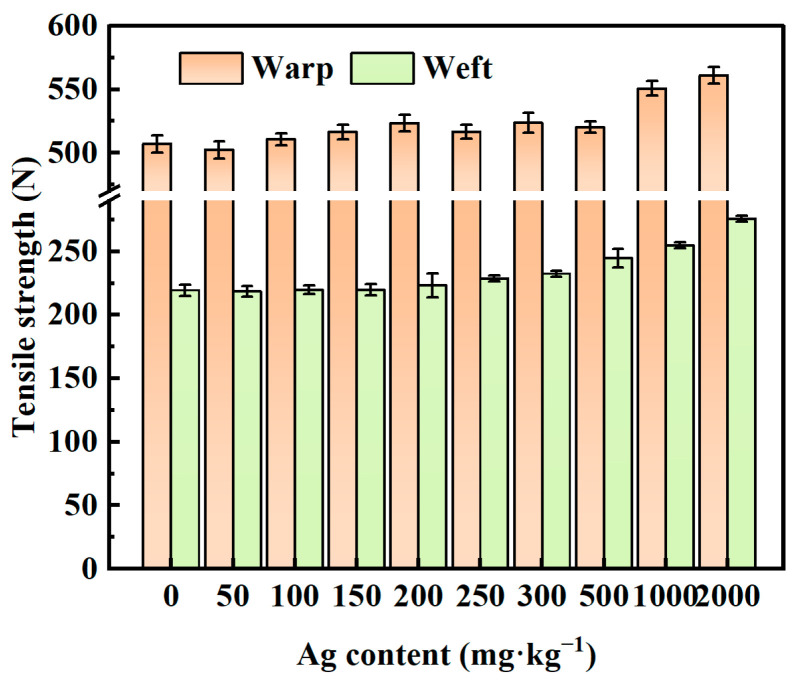
Tensile properties of the virgin polyester fabric and PDMS/Ag@PET fabrics.

The tensile test results depicted in Figure 11 reveal that as the silver content increased, the breaking strength of the fabrics gradually increased. Furthermore, the mechanical properties of the polyester fabrics increased following plasma crosslinking. In particular, compared with those of the original polyester fabrics, the warp and weft breaking strengths of the polyester fabrics with a silver content of 2000 mg/kg improved by 10.6% and 25.7%, respectively. This enhancement can be attributed to the presence of the PDMS coating, which induced greater adhesion between the fibers. Additionally, PDMS molecules penetrated the amorphous zone of the fibers, thereby increasing the cohesion of the fabric and preventing fiber slippage, ultimately contributing to the improved breaking strength of the fabric.

The comfort of worn fabrics is closely related to their moisture, air, and water permeability resistance, which is further influenced by the fabric structure, such as its pore size and fiber surface properties. The properties of the original polyester, PDMS@PET, and PDMS/Ag@PET fabrics with a nanosilver loading of 300 mg/kg were tested, and the results are presented in Table 2.

The air permeability of the original polyester fabric was approximately 1648.47 mm·s^−1^. Following the plasma impregnation crosslinking treatment, there was a slight, though not significant, decrease in air permeability, with PDMS@PET and PDMS/Ag@PET experiencing reductions of 0.64% and 1.0%, respectively. Additionally, as demonstrated in Video S3, the PDMS/Ag@PET fabric was placed over a glass bottle containing ammonia with hydrochloric acid nearby, and it was observed that the volatility of the two substances caused white fumes from the reaction to fill the air. The reaction equation is NH_3_ + HCl = NH_4_Cl. This observation indirectly suggests that the formation of a thin film on the fabric surface using the plasma method does not completely block the pores between the fabric yarns. Instead, the crosslinking of PDMS occurs mainly at the fiber surface, resulting in fabrics with excellent air permeability.

On the other hand, the moisture permeability of the polyester fabric decreased more significantly after plasma hydrophobic treatment. The original polyester fabric exhibited a moisture permeability of approximately 5311.66 g/(m^2^·24 h). Following treatment, both PDMS@PET and PDMS/Ag@PET experienced moisture permeability reductions of 29.86% and 28.42%, respectively. In fact, the PDMS coating had a more significant effect on the void spaces within the polyester fabrics, leading to a decrease in moisture permeability. This decrease can be attributed to a combination of factors, including a decrease in fabric water absorption and porosity, ultimately resulting in increased water permeability pressure or hydrostatic pressure. Consequently, after plasma hydrophobic treatment, the water permeability pressures of the PDMS@PET and PDMS/Ag@PET fabrics increased by 39% and 59%, respectively, indicating improved water permeability resistance.

## 3. Experimental Section

### 3.1. Materials

Silver nitrate (AgNO_3_) was procured from the Shanghai Institute of Fine Chemical Materials (Shanghai, China). Water-soluble polyurethane (anionic aliphatic polyether type) with a viscosity exceeding 250 mPa·S at 25 °C and a solid content of 38 ± 2%, maintaining a pH of 6–7 at 25 °C, was obtained from Qiancheng Plastic Chemical Materials Co., Ltd. (Qingdao, China). Sodium borohydride (NaBH_4_) and ammonia were obtained from Sinopharm (Beijing, China). Gram-negative *Escherichia coli* (*E. coli*, ATCC 25922) and Gram-positive *Staphylococcus aureus* (*S. aureus*, CMCC26003) were obtained from Shanghai Luwei Technology Co., Ltd. (Shanghai, China). Nutrient agar and nutrient broth were acquired from Shanghai Zhongke Insect Biotechnology Development Co., Ltd. (Shanghai, China). Phosphate buffer was purchased from Shanghai Bowei Biotechnology Co., Ltd. (Shanghai, China). PDMS (CAS#: 63148-62-9, kinematic viscosity: 10 cSt at 25 °C, molar mass: 74.15 g/mol) was obtained from Jiangnan Jiashan Textile Materials Co., Ltd. (Jiaxing, China). Detergent 209 was procured from Wangnilai Co., Ltd. (Guangzhou, China). The polyester fabrics (100%, 120 g/m^2^) were obtained from Miandu Textile Co., Ltd. (Yiwu, China). Deionized water was prepared in the laboratory.

### 3.2. Preparation and Characterization of Emulsions

PDMS–water emulsion: A specific quantity of PDMS was directly introduced into 500 mL of deionized water. This mixture was subsequently processed to yield PDMS–water emulsions with varying concentrations, ranging from 1 wt.% to 20 wt.%, through high-speed shearing (~5000 rpm) utilizing a homogenizing emulsification machine (JRH-1000 Emulsifying Machine, Hangzhou Qiwei Instrument Co., Ltd., Hangzhou, China).

PDMS/Ag–water emulsion: The preparation of silver nano-solutions followed an improved protocol based on previously reported methods [38]. Initially, 404 mL of a solution of silver nitrate at a concentration of 7.8 g/L was thoroughly stirred in a thermostatic oscillator (THZ-C, Suzhou Peiying Experimental Equipment Co., Ltd., Suzhou, China) until complete dissolution was achieved. Subsequently, ammonia was added dropwise until the solution became transparent, causing the solution to change from colorless to dark brown and resulting in a clarified and transparent silver–ammonia solution. Next, a specific quantity of aqueous polyurethane solution with a solid content of 30% was added dropwise as a protective agent (with a ratio of 6:1 to 9:1 between the aqueous polyurethane content and the silver monomer content). This mixture was stirred for 30 min. Afterwards, a 1 g/L sodium borohydride solution was slowly introduced to facilitate the chemical in situ reduction of silver nitrate. The final reaction mixture was adjusted to a volume of 500 mL and stored in the dark. The nanosilver solution prepared in the aforementioned steps was added to a specified mass of deionized water and diluted to achieve a solution with a silver concentration ranging from 50 mg/L to 2000 mg/L. Subsequently, PDMS was incorporated into the solution, and the resulting mixture was emulsified using high-speed homogenizing shear for 30 min. Ultimately, the PDMS/Ag emulsion was prepared as a fully aqueous system.

### 3.3. Preparation of Superhydrophobic Antimicrobial Fabrics

Initially, a solution containing 2 g/L detergent 209 and 2 g/L sodium carbonate was prepared. Subsequently, the PET fabrics were immersed in this solution to eliminate any potential dust or chemical residues. The immersion was conducted in an ultrasonic bath with a bathing ratio of 40:1 for a duration of 30 min. Following this step, the fabric was rinsed with deionized water approximately four to five times until no detergent residue remained. Subsequently, the fabric was dried at 60 °C and set aside [49].

The dried fabrics were then subjected to impregnation in both PDMS–water and PDMS/Ag–water emulsions, each for a period of 30 min, with the pick-up rate thoroughly controlled at 100%. After impregnation, the fabrics were dried at 60 °C and subsequently introduced into plasma discharge equipment (AP600 type, Nordson-March corporation, Concord, CA, USA) for plasma-induced crosslinking treatment. This process led to the creation of superhydrophobic polyester fabrics, denoted as PDMS@PET, and superhydrophobic antibacterial polyester fabrics, referred to as PDMS/Ag@PET, with nanosilver loadings ranging from 50 mg/kg to 2000 mg/kg. The parameters for the plasma crosslinking process were derived from a prior investigation [34], including a power setting of 100 W, discharge pressure of 220 Pa, treatment time spanning 60 s, and working gas Ar.

### 3.4. Characterization

The dispersion and morphological structure of the emulsions were examined using transmission electron microscopy (TEM; JEM-2100, JEOL, Tokyo, Japan). The stability of the PDMS/Ag–water emulsion was assessed by analyzing the particle size and zeta potential using a nanoparticle analyzer (90Plus PALS, Brookhaven Instruments Corporation, Holtsville, NY, USA). To investigate the surface morphology of the fabrics, scanning electron microscopy (SEM; ZEISS Gemini SEM300, Carl Zeiss Company, Oberkochen, Germany) was employed, with an accelerating voltage set at 15 kV. The chemical components on the fabric surfaces were analyzed using Fourier transform infrared spectroscopy (FTIR; Thermo Nicolet iS50, Thermo Fisher Scientific, Waltham, MA, USA) and X-ray photoelectron spectroscopy (XPS; Thermo Scientific K-Alpha+, Waltham, MA, USA). Furthermore, the thermal stability of the fabrics was assessed through thermogravimetric analysis (TG; TG209F3, NETZSCH, Selb, Germany). The heating process involved increasing the temperature from 30 °C to 900 °C at a heating rate of 40 °C/min under a nitrogen atmosphere. The WCA of the fabrics was determined using a contact angle measuring instrument (OCA15EC, Dataphysics, Filderstadt, Germany). A 5 μL droplet of deionized water was used as the test sample at room temperature, with a water outflow rate of 2 μL/s. The average WCA was obtained by measuring five different points on each piece of fabric.

### 3.5. Mechanical Durability and Acid and Alkali Resistance Test

The mechanical durability of the fabric surface was assessed based on two aspects: washing stability and abrasion resistance. For washing stability, a color fastness washing tester (SW-12J, Wenzhou Darong Textile Instrument Co., Ltd., Wenzhou, China) was based on GB/T 3921-2008 [52]. The washing procedure involved the use of a solution consisting of standard soap flakes in deionized water at a concentration of 5 g/L. The bathing ratio was maintained at 50:1, the rotational speed was 40 ± 2 r/min, the washing temperature was 40 °C, and the duration was 30 min. Subsequently, the fabrics were rinsed with deionized water and subsequently dried at 60 °C. To assess the washing durability, the WCA and antimicrobial properties were measured after the washing treatment. For abrasion resistance testing, a dyeing and rubbing fastness machine (Y571N, Nantong Hongda Instrument Co., Ltd., Nantong, China) was used in accordance with GB/T 3920-2008 [53]. The samples were initially cut to dimensions not less than 200 mm × 50 mm and were required to be clean and flat and devoid of any folds or tears. The applied operating pressure was 44.8 kPa, and the abrasive head consisted of white standard cotton lining. The rubbing speed was set at 60 cycles/min. After completion of the test, the abrasion resistance of the fabric surface was evaluated by performing the WCA test for varying numbers of abrasion cycles. To assess the fabric’s response to different pH environments, acidic and alkaline solutions with different pH values were prepared using NaOH and HCl. The superhydrophobic antimicrobial fabrics were immersed in these solutions for 12 h, after which their WCAs were subsequently measured to evaluate their performance under different pH conditions.

### 3.6. Antimicrobial Performance Test

The antimicrobial efficacy of the PDMS/Ag@PET fabrics was assessed according to the enhanced GB/T 20944.3-2008 [54]. The test strains included Gram-negative *Escherichia coli* and Gram-positive *Staphylococcus aureus*. Before each test, the samples, glassware, nutrient broth, silver phosphate buffer, and nutrient agar utilized in the experiment were sterilized in an autoclave at 121 °C and 103 kPa for 20 min. These items were subsequently set aside.

A small quantity of bacteria was cultured in 20 mL of nutrient broth and placed in a 37 °C constant-temperature oscillator at 80 r/min for 18–24 h. The resulting bacterial solution was subsequently diluted to a concentration of 3–5 × 10^5^ CFU/mL using both nutrient broth and 0.03 mol/L phosphate-buffered solution (PBS) with a pH range of 7.0–7.2. For the antimicrobial test, 3 mL of the diluted bacterial solution was brought into contact with 0.1 g of the fabric sample, and the mixture was incubated for 18–24 h at 80 r/min in a constant-temperature shaker at 37 °C. Following incubation, 15 mL of nutrient agar was poured into a Petri dish and allowed to cool and solidify. Subsequently, 100 μL of the bacterial solution that had been incubated with the fabric sample was extracted, serially diluted with 0.03 mol/L phosphate buffer (×10^1^, ×10^2^, ×10^3^, and ×10^4^), and subsequently transferred to nutrient agar. The fabrics were then incubated in a biochemical incubator at 37 °C for 18–24 h. Polyester prototype fabrics were used as control samples to calculate the bacterial inhibition rate. The formula for calculating the bacterial inhibition rate was as follows:

$$Inhibitionrate=\frac{B-A}{B}\times 100\%$$

where B is the number of colonies in the control sample Petri dish and A is the number of colonies in the test sample Petri dish.

### 3.7. Self-Cleaning Performance

A small quantity of solid powder, including chalk dust and fine sand, was sporadically scattered onto the surfaces of the pristine PET, PDMS@PET, and PDMS/Ag@PET fabrics. These fabrics were subsequently securely placed against a slide at an inclination of approximately 20°. The self-cleaning performance of the water droplets rolling off the fabric surfaces was evaluated.

### 3.8. Wetting Resistance Test

The evaluation of fabric water repellency primarily involved assessing the spray wetting level and hydrostatic pressure resistance. To test the waterproofing performance of the different fabrics, a spray device (AATCC22, Ningbo Textile Instrument Co., Ltd., Ninbo, China) was used following the guidelines specified in AATCC test method 22-2005 [55]. Spray rating refers to how well a fabric surface resists being wetted by water. Spray ratings range from zero to five; the higher the number, the better the water resistance. Additionally, a digital water permeability tester (YG (B) 812D-20, Wenzhou Darong Textile Instrument Co., Ltd., Wenzhou, China) was utilized to evaluate the water permeability performance of the samples, in accordance with the standard AATCC 127-2017 [56]. During the test, the surface of the fabric was brought into contact with water via the pressurization method, during which the water pressure increased at a rate of 500 mmH_2_O/min. Observations were continuously made to detect any signs of water penetration. The water seepage pressure of the sample was determined by recording the pressure at which water seeped in three different locations. Each sample was tested five times, after which the average value was calculated.

### 3.9. Tensile Property Test

Following the GB/T 3923.1-2013 [57] guidelines, the tensile breaking strength of the cotton fabric was assessed using a multifunctional electronic fabric strength machine (YG026MB, Wenzhou Fangyuan Instrument Co., Ltd., Wenzhou, China). The fabrics were cut along both the warp and weft directions, resulting in sample dimensions of 30 cm × 5 cm. The spacing length between grips was set at 10 cm, and the tensile speed was maintained at 100 cm/min. The tensile breaking strength of the fabrics was measured and recorded five times, after which the average value was subsequently calculated.

### 3.10. Comfort Wearing of the Fabric

The wearing comfort of fabrics relies on both their air permeability and moisture permeability. To assess the effect of superhydrophobic antimicrobial treatment on the wearing comfort of various polyester fabrics, different tests were conducted. In accordance with GB/T 5453-1997 [58], test fabrics measuring 250 mm × 250 mm were cut and securely clamped onto the testing bench. The air pressure was set at 100 Pa, nozzle No. 06 was selected, and testing conditions were established at a temperature of 25 °C with a humidity level of 60%. Five tests were performed on each fabric specimen to determine the average air permeability. Additionally, following the standards outlined in GB/T12704.2-2009 [59], the moisture permeability of the fabrics was assessed using a Fabric Water Vapor Permeability Tester (YG(B)216 G, Wenzhou Darong Textile Instrument Co., Ltd., Wenzhou, China).

## 4. Conclusions

In summary, this study successfully developed a robust and durable superhydrophobic antibacterial PDMS/Ag@PET fabric through a green, environmentally friendly, straightforward, and practical aqueous treatment method. Remarkable superhydrophobicity and antimicrobial properties were achieved when the PDMS concentration reached 5 wt.% and the nanosilver content was 50 mg/kg or higher. The combination of a micron-scale pleated structure and low surface energy allowed the polyester fabrics to reach a WCA of 154.2°, and the presence of nanosilver conferred a 99.99% inhibition rate against *E. coli* and *S. aureus*. Furthermore, the prepared PDMS/Ag@PET fabrics demonstrated/resistance to washing and abrasion when subjected to multiple cycles of mechanical stress. These fabrics, manufactured using this experimental procedure, maintained physical properties akin to those of the original polyester fabrics, including air permeability and breaking strength. This production method holds promise for expanding the production of multifunctional fabrics and unlocking their potential in the field of microbial protective textiles for medical, domestic, and industrial applications.

## Figures and Tables

**Table 1:** *E. coli* and *S. aureus* were inhibited after 100 standard washes.

Ag Content (mg·kg^−1^)	Antibacterial Rates (%)	
	*E. coli*	*S. aureus*
50	90.99	91.99
100	98.89	96.97
150	99.99	99.99
200	99.99	99.99
250	99.99	99.99
300	99.99	99.99

**Table 2:** Air permeability, moisture permeability, and water impermeability of PET, PDMS@PET, and PDMS/Ag@PET fabrics.

Sample	Air Permeability (mm/s)	Moisture Permeability (g/(m^2^·24h))	Seepage Pressure (kpa)
PET	1648.47	5311.66	0.02
PDMS@PET	1637.87	3725.8	0.8
PDMS/Ag@PET	1632.73	3802.12	1.2

## Data Availability

Data are contained within the article and Supplementary Materials.

## Supplementary Materials

The following supporting information can be downloaded at: https://www.mdpi.com/article/10.3390/molecules29061219/s1, Figure S1: (a) optical photograph of PDMS dispersion in water, (b) zeta potential of PDMS emulsion, (c,d) particle size distribution of PDMS emulsion before and after standing for half an hour; Figure S2: (a) wetting in water of pristine polyester fabric (left) and plasma etched polyester fabric (right), (b) XPS and (c,d) the high-resolution C 1s spectra of pristine and plasma etched polyester fabrics; Figure S3: the WCA of PDMS/Ag@PET fabrics varied with the content of nano silver without PDMS; Figure S4: SEM and AFM images of PDMS@PET fabrics with PDMS concentrations of (a,d) 1 wt.%, (b,e) 5 wt.%, (c,f) 15 wt.%; Figure S5: variation of WCA with PDMS concentration in ethanol solution; Figure S6: photographs of (a) *E. coli* and (b) *S. aureus* colonies on the nutrient agar of PDMS@PET fabrics; Table S1: the elemental content changes of polyester fabrics before and after plasma etching; Video S1: spray wettability; Video S2: self-cleaning process of PET, PDMS @PET fabrics, and PDMS/Ag@PET fabrics; Video S3: the air permeability of PDMS/Ag@PET.
