# Supplementary material for: Green Method for the Preparation of Durable Superhydrophobic Antimicrobial Polyester Fabrics with Micro-Pleated Structures
Source: Molecules. 2024 Mar 8;29(6):1219. doi: 10.3390/molecules29061219 (PMC10974891; doi:10.3390/molecules29061219)
Supplement: Supplementary file 1 [file molecules-29-01219-s001.zip › molecules-2871546-supplementary.pdf]

SUPPLEMENTARY MATERIALS

Green Method for the Preparation of Durable Superhydrophobic Antimicrobial Polyester Fabrics with Micro-Pleated Structures

Ying Zhao [1], Kaihong Chen [1], Jiehui Zhu [1], Huajie Chen [1], Yong Xia [1], Minglin Xu [2], Liyun Xu [1,3,*] and Lirong Yao [1,3,*]

1 College of Textile and Clothing, Nantong University, Nantong 226019, China
2 Langfang Feize Composites Technology Co., Ltd., Langfanng 065003, China
3 National & Local Joint Engineering Research Center of Technical Fiber Composites for Safety and Protection, Nantong University, Nantong 226019, China
* Correspondence: lyxu@ntu.edu.cn (L.X.); ylr8231@ntu.edu.cn (L.Y.)

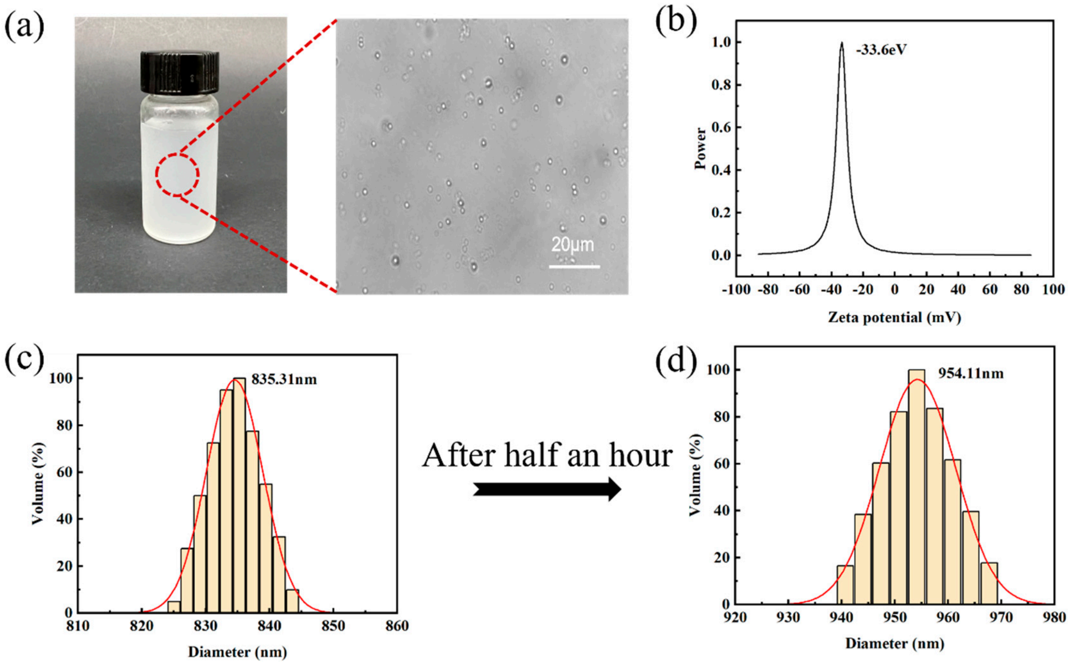

Figure S1. (a) Optical photograph of polydimethylsiloxane (PDMS) dispersion in water. (b) Zeta potential of PDMS emulsion. (c–d) Particle size distribution of PDMS emulsion before and after standing for half an hour.

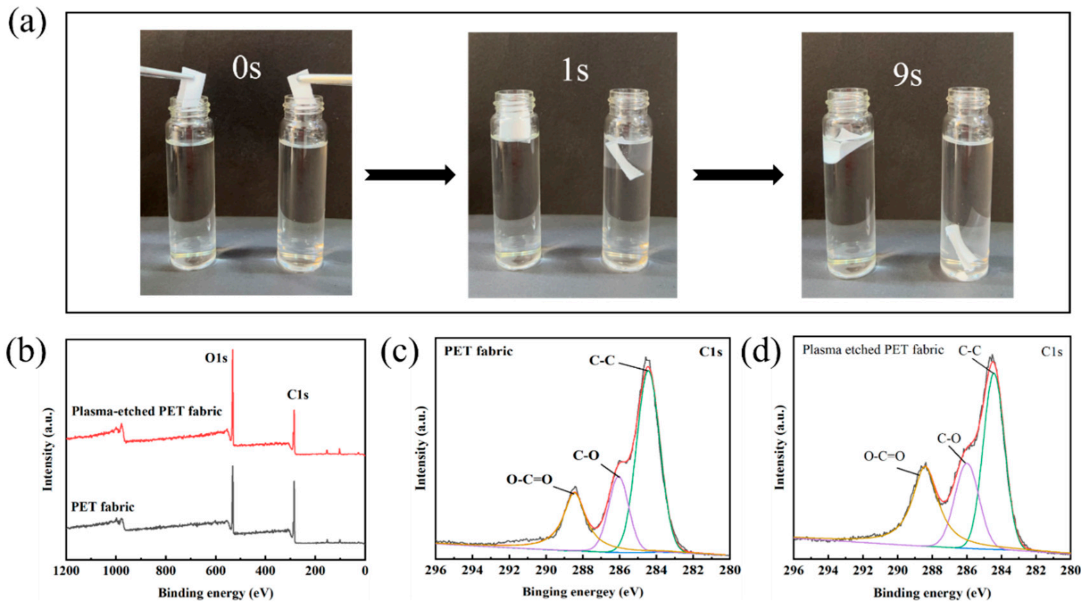

Figure S2. (a) Wetting in water of pristine polyester fabric (left) and plasma etched polyester fabric (right). (b) X-ray photoelectron spectrometry (XPS) and (c–d) the high-resolution C 1s spectra of pristine and plasma-etched polyester fabrics.

Table S1. The elemental content changes of polyester fabrics before and after plasma etching.

Sample	Element content (At.%)		Surface group content (%)		
	C	O	C-C	C-O	C=O
Pristine PET	69.77	29.14	53.89	23.22	22.89
Plasma-etched PET	59.14	39.24	43.26	22.39	34.34

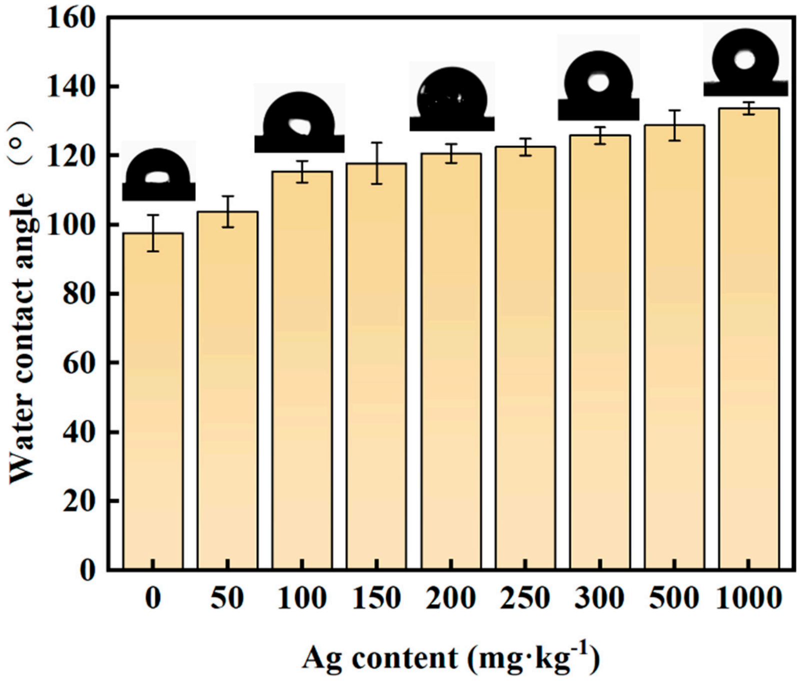

Figure S3. The water contact angle (WCA) of PDMS/Ag@PET fabrics (superhydrophobic antibacterial polyester fabrics) varied with the content of nanosilver without PDMS.

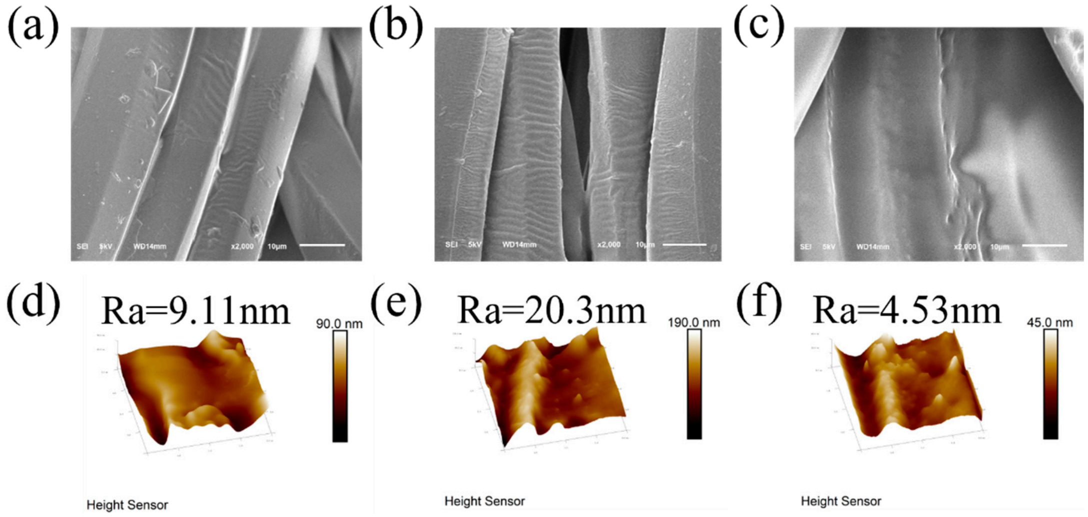

Figure S4. Scanning electron microscope (SEM) and atomic force microscope (AFM) images of PDMS@PET (superhydrophobic polyester fabrics) fabrics with PDMS concentrations of (a, d) 1 wt.%, (b, e) 5 wt.%, and (c, f) 15 wt.%.

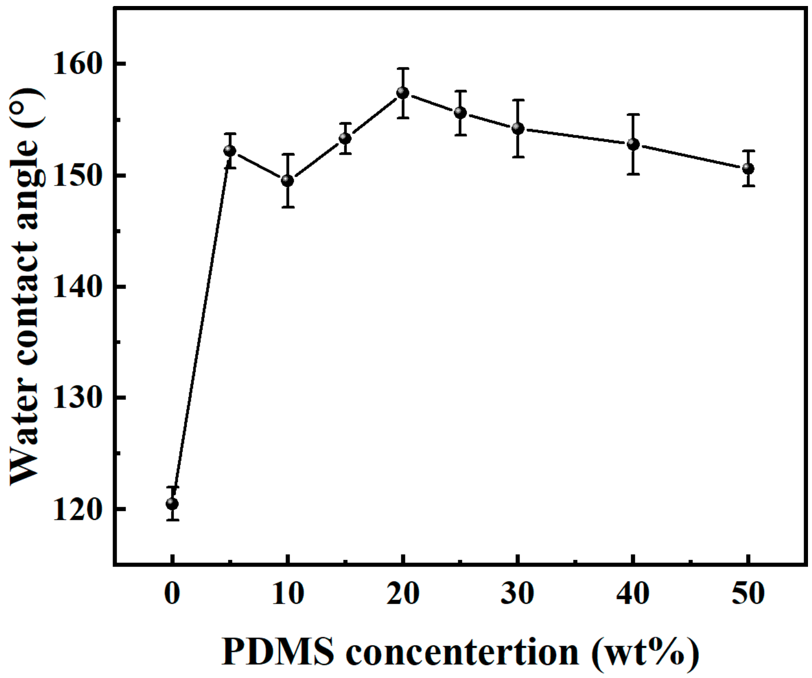

Figure S5. Variation of WCA with PDMS concentration in ethanol solution.

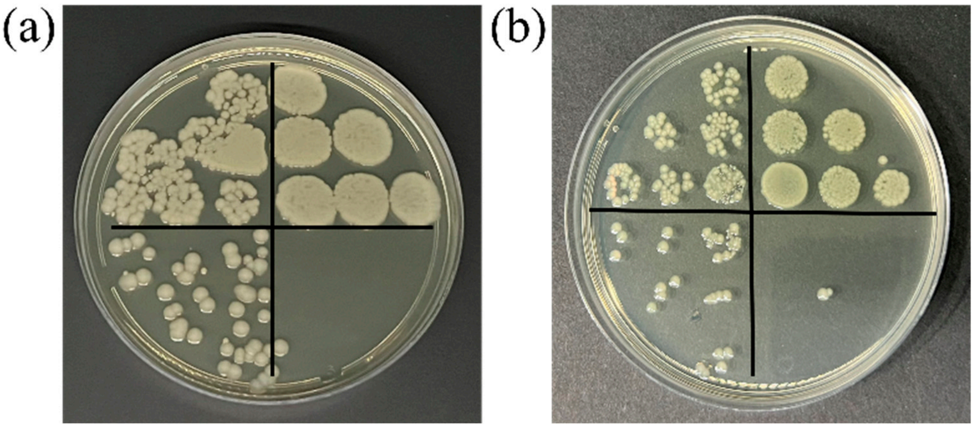

Figure S6. Photographs of (a) E. coli and (b) S. aureus colonies on the nutrient agar of PDMS@PET fabrics.

Video S1. Spray wettability.

Video S2. Self-cleaning process of PET, PDMS @PET fabrics, and PDMS/Ag@PET fabrics.

Video S3. The air permeability of PDMS/Ag@PET fabrics.
